# Automated Processing and Evaluation of Anti-Nuclear Antibody Indirect Immunofluorescence Testing

**DOI:** 10.3389/fimmu.2018.00927

**Published:** 2018-05-04

**Authors:** Vincent Ricchiuti, Joseph Adams, Donna J. Hardy, Alexander Katayev, James K. Fleming

**Affiliations:** ^1^North Central Division, Laboratory Corporation of America Holdings (LabCorp), Dublin, OH, United States; ^2^Department of Science and Technology, Laboratory Corporation of America Holdings (LabCorp), Elon, NC, United States

**Keywords:** anti-nuclear antibodies, autoimmune rheumatic diseases, automation, computer-aided immunofluorescence microscopy, EUROPattern Suite, HEp-20-10 cells, indirect immunofluorescence, standardization

## Abstract

Indirect immunofluorescence (IIF) is considered by the American College of Rheumatology (ACR) and the international consensus on ANA patterns (ICAP) the gold standard for the screening of anti-nuclear antibodies (ANA). As conventional IIF is labor intensive, time-consuming, subjective, and poorly standardized, there have been ongoing efforts to improve the standardization of reagents and to develop automated platforms for assay incubation, microscopy, and evaluation. In this study, the workflow and performance characteristics of a fully automated ANA IIF system (Sprinter XL, EUROPattern Suite, IFA 40: HEp-20-10 cells) were compared to a manual approach using visual microscopy with a filter device for single-well titration and to technologist reading. The Sprinter/EUROPattern system enabled the processing of large daily workload cohorts in less than 8 h and the reduction of labor hands-on time by more than 4 h. Regarding the discrimination of positive from negative samples, the overall agreement of the EUROPattern software with technologist reading was higher (95.6%) than when compared to the current method (89.4%). Moreover, the software was consistent with technologist reading in 80.6–97.5% of patterns and 71.0–93.8% of titers. In conclusion, the Sprinter/EUROPattern system provides substantial labor savings and good concordance with technologist ANA IIF microscopy, thus increasing standardization, laboratory efficiency, and removing subjectivity.

## Introduction

Anti-nuclear antibodies (ANA) represent important diagnostic markers in various autoimmune rheumatic conditions (e.g., systemic lupus erythematosus (SLE), Sjögren’s syndrome, systemic sclerosis, dermato/poly myositis, mixed connective tissue diseases, and rheumatoid arthritis), with an increasingly recognized relevance to disease prediction and prognosis (1–6). Low-titer ANA may also be detected in healthy individuals (7–9). The term “ANA” is commonly used *sensu lato* to encompass not only antibodies directed against nuclear antigens, but also those binding to constituents of the nuclear envelope, mitotic spindle apparatus, or cytoplasm.

In 1957, the first ANA was demonstrated by indirect immunofluorescence (IIF) in the serum of SLE patients, followed by the discovery and characterization of extractable nuclear antigens in 1959 (10–12). IIF testing has since become the standard method for ANA screening in patient sera, using human epithelial cells (HEp-2) or variants of this laryngeal carcinoma cell line as the preferred cell substrate (13, 14). Hep-2 cells present a very broad spectrum of 100–150 cell antigens at different stages of the cell cycle, allowing the sensitive detection of numerous clinically relevant autoantibodies. However, conventional ANA IIF testing is time-consuming, laborious, and burdened by the need for micro-scopy expertise, subjectivity of interpretation, lack of automation, and a low degree of standardization leading to high intra- and inter-laboratory variance (15–18). As the demand for ANA testing has increased considerably over the past decades and pushed large service laboratories to provide high throughput, reduced turnaround time-consuming and cost-saving diagnostics, there has been a movement from IIF to largely automated screening methods, in particular ELISA and flow cytometric bead-based (“multiplex”) immunoassays that are based on a limited number of purified and/or recombinant antigenic substrates. Examples for multiplex assays include the BioPlex 2200 ANA screen(Bio-Rad), Athena Multi-Lyte (ZEUS Scientific), Quanta Plex (INOVA Diagnostics), and FIDIS (BMD) (13, 16, 19–30). Samples classified as positive through screening by ELISA or multiplex are usually reflexed to IIF to confirm the result and to determine the titer and associated ANA pattern(s), while samples devoid of reactivity against the antigenic panel are reported as negative. Although this approach is time-consuming and cost-saving and provides a high specificity for each single antigen, the use of screening panels has slightly less sensitivity than HEp-2-based IIF. In 2007, the American College of Rheumatology setup a task force which soon after released a position statement recommending IIF as the “gold standard” for ANA testing (13, 31). This concept was adopted later by international organizations and, along with advances in IIF automation, led to a “renaissance” of IIF (16, 32). In current practice, a two-step strategy is commonly applied, where initial ANA IIF screening provides information on antibody patterns and titers, followed by a confirmatory monospecific test (e.g., ELISA, Multiplex, and immunoblot) to identify the autoantibody (33), or in many laboratories, the reverse algorithm is also performed, where enzyme immunoassay positivity is reflexed to IIF.

In 2015, the persisting lack of inter-laboratory standardization and other problems in ANA IIF testing and reporting put forth an International Consensus on ANA patterns (ICAP) (34, 35). Beside the main objective of (i) standardizing the categorization and nomenclature of HEp-2 cell ANA patterns, the ICAP consensus also recommended (ii) endpoint titration of positive samples. The relevance of this point becomes clear considering that single-well testing of high-titer sera bears the risk of antibody masking. Masking may occur when a diagnostically relevant autoantibody is indiscernible due to the presence of further dominant or unspecific antibodies or when hook/prozone effects from antibody excess cause atypical, diffuse, faint, or negative IIF staining (36, 37). (iii) Clinically relevant mixed patterns should be discriminated accurately considering the possibility of antibody masking. (iv) The ICAP intention is to differentiate patterns that should be readily recognized (competent-level) from patterns that would be more challenging and distinguishable only when observers or technologists have attained a expert-level proficiency. Reporting should include all competent-level nuclear and cytoplasmic patterns. Optimally, all patterns seen in a positive sample should be reported regardless of the clinical relevance. (v) Transfected HEp-2 cells for general pattern definition should not be used.

Additionally, the biomedical industry has improved IIF standardization for the preparation of substrates and slides, the automation of slide incubation, software-based image acquisition and interpretation (computer-aided IF microscopy) (38–44), as well as the automated transfer of results. Different commercial systems for automated ANA IIF testing have recently been developed and evaluated (17, 29, 30, 45–55). The Sprinter XL and the EUROPattern Suite (Euroimmun, Lübeck, Germany) have been designed to provide a high-throughput platform for automated specimen processing and slide incubation as well as for automated microscopy, titer, and pattern interpretation (23, 56).

In this study, the ANA IIF workflow characteristics and analytical performance of the Sprinter/EUROPattern system were evaluated against manual processing and visual microscopy with or without titration support.

## Methods

### Human Sera

Analysis of the workflow and of labor savings was calculated on consecutive serum samples representing the daily workload cohorts for routine ANA analysis at the respective laboratories within Laboratory Corporation of America^®^ Holdings (LabCorp) reference laboratory network in the USA. For the evaluation of assay performance, we used 97 ANA negative samples and 176 patient samples pre-characterized as ANA positive using the PolyTiter immunofluorescence system (Polymedco, Inc., Cortland Manor, NY, USA) and ANAFLUOR Hep-2 reagent kit (DiaSorin, Stillwater, MN, USA). These samples had been sent to the LabCorp Dublin Regional Laboratory (OH) for routine antibody screening. The cohort of 176 positive samples was grouped according to the ANA pattern detected by the manual protocol [68 homogeneous, 41 granular (speckled), 20 centromere, 22 nucleolar, and 25 mixed patterns]. In accordance with the Declaration of Helsinki (1964) ethical guidelines, samples were blinded for analysis to maintain confidentiality. The study protocol was determined to be exempt by the Institutional Review Board (Western Institutional Review Board^®^, Puyallup, WA, USA).

### Data Collection

Workflow data were collected at LabCorp laboratories in Birmingham (AL), Burlington (NC), Dallas (TX), Dublin (OH), Houston (TX), Phoenix (AZ), Raritan (NJ), and Tampa (FL). Analyses to evaluate diagnostic performance were performed in Dublin LabCorp Laboratory.

### IIF Testing for ANA Using Three Approaches

#### Current Method: ANAFLUOR Hep-2 Reagent Kit (DiaSorin, Stillwater, MN, USA)

Sample preparation and slide incubation were processed according to the manufacturer’s procedure. Only one serum dilution (1:40) is required for the titer determination. Each processed slide was read independently under fluorescent microscopy by three experienced technologists complemented with an immunofluorescent titration system: PolyTiter immunofluorescence system (Polymedco, Inc., Cortland Manor, NY, USA). The latter uses filter-controlled light attenuation to determine semiquantitatively the ANA endpoint titer from a single serum dilution by relating the intensity of fluorescent staining to reference calibrators. The titration system is comprised of hardware (digital control pad, filter unit, and microscope adapters), software, and pre-diluted calibrator solutions with endpoint titer values of 1:40, 1:160, 1:640, and1:2,560. Diluted patient sera, kit controls, and calibrators are assayed according to the manufacturer’s instructions. Results for ANA patterns and calculated titers were entered into laboratory information system (LIS) and reviewed manually by two technologists.

#### Conventional Visual IIF Microscopy

If PolyTiter results were questionable, sample preparation and slide incubation were performed manually using the ANAFLUOR Hep-2 reagent kit. Serum sample titers were manually prepared using the following dilutions: 1:40, 1:80, 1:160, 1:320, 1:640, and 1:1,280. Experienced technologists using fluorescent microscopy read each slide independently, and results were entered manually.

#### Automated ANA IIF Protocol: Sprinter/EUROPattern System

**Figure 1:**
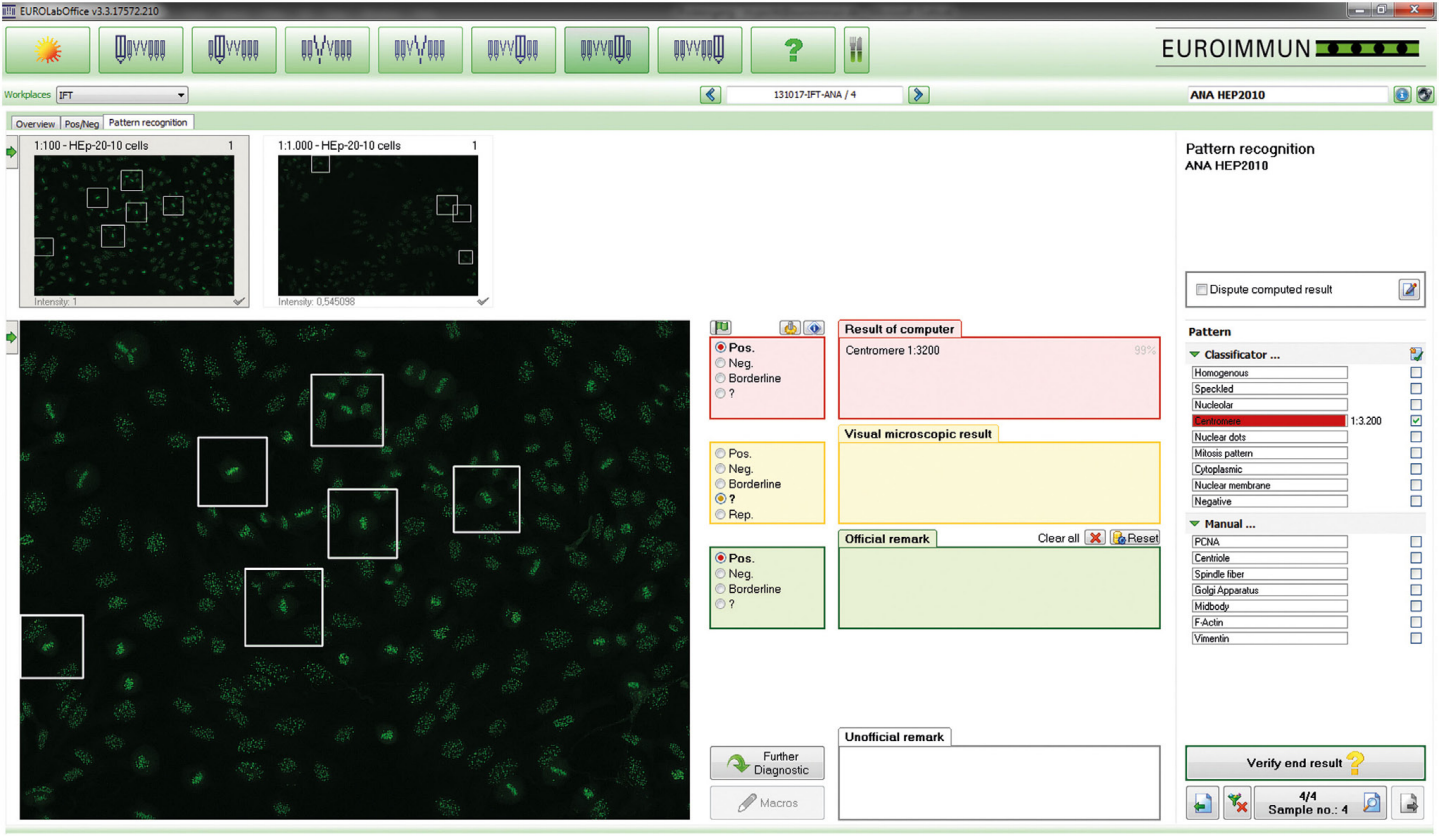
EUROPattern Suite graphical user interface. For each sample classified as positive, the system displays the indirect immunofluorescence (IIF) images for different dilutions/substrates (left) and the proposed results of automated interpretation (right), merging all proposed results (classification, titer, confidence value) into one report per patient. To support pattern interpretation, mitotic cells and late metaphase chromosomes can be highlighted. The proposed results are to be confirmed (or modified) interactively by the technologist. Negative results are displayed in a small-format scroll-down register and can be verified batch-wise (data not shown).

Samples and slides were processed using the Sprinter XL (Euroimmun AG, Lübeck, Germany), followed by automated evaluation using the EUROPattern Suite (Euroimmun AG, Lübeck, Germany). ANA detection was performed by the IFA 40: HEp-20-10 kit assay according to the manufacturer’s instructions (Euroimmun AG, Lübeck, Germany) (57). The IFA 40: HEp-20-10 allows for easier evaluation due to an increased spectrum of cells in the mitotic phase and of human nuclear antigens when compared to traditional Hep-2 cells. The increase of cells in the mitotic phase offers easy confirmation of reactions (Figure 1). The slides have 10-reaction fields, each containing a biochip coated with HEp-20-10 cells. Serum samples were diluted in PBS-Tween and screened for ANA at 1:40. Positive samples (1:80 and above) were reflexed for titers at three dilutions (1:80, 1:320, and 1:1,280) by automated dilution using Sprinter XL system. The fluorescein isothiocyanate-labeled anti-human IgG conjugate solution contains propidium iodide as counterstain for image segmentation by EUROPattern.

**Figure 2:**
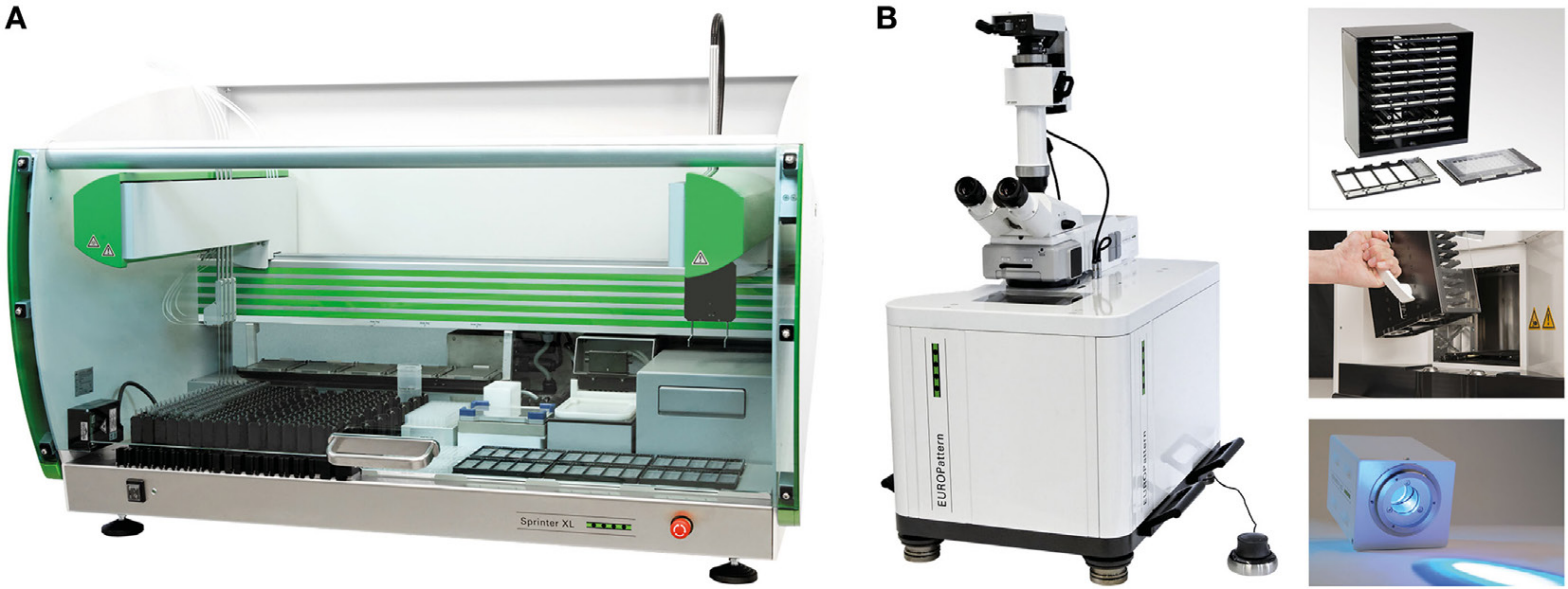
Automation of anti-nuclear antibodies indirect immunofluorescence (IIF). **(A)** Sprinter XL for fully automated processing of IIF tests from the dilution and dispensing of samples to the incubation and washing of microscope slides. In addition, the system is capable of running ELISA microplates. Capacity: 160–240 sample tubes, 30 ten-field IIF slides, six microplates. **(B)** The EUROPattern Suite microscope is equipped, among others, a customized set of autofocusing objectives (10×/20×/40×), oculars (optional), two high-resolution cameras, long-life cLED, 3D manual controller, matrix code reader, and slide magazine plus carrier. Capacity: 50 ten-field slides (500 analyses), 18 sec/analysis.

**Figure 3:**
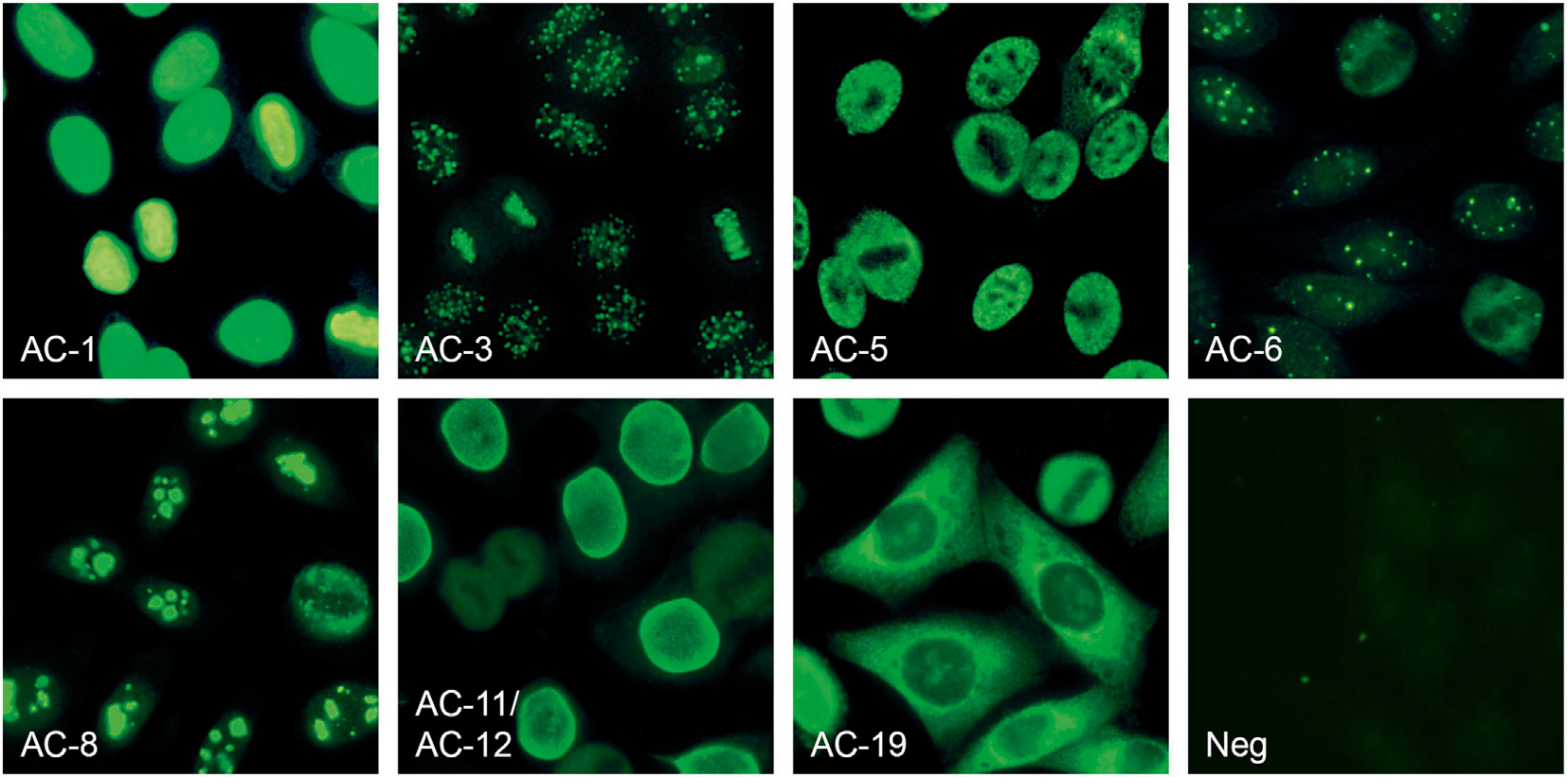
Anti-nuclear antibodies (ANA) patterns recognized by the EUROPattern Suite software (numbering according to International consensus on ANA patterns): homogeneous (AC-1), centromere (AC-3), speckled (AC-5), nuclear dots (AC-6), nucleolar (AC-8), nuclear membrane (AC-11, AC-12), cytoplasmic (AC-19), and negative/unspecific (Neg). Mixed patterns with varying titers can also be identified (data not shown).

The Sprinter XL provides automated processing of IIF tests, including sample identification, dilution, and dispensing, followed by slide incubation and washing (Figure 2A). The Sprinter XL system has a loading capacity of up to 240 samples and 30 slides, which are identified through barcode and matrix code readers, respectively. The pipetting unit comprises two arms and four washable needles. Washing is based on slide flooding. The EURO-Pattern Suite is a system to record, evaluate, and archive digital images of IIF slides. It is based on a combination of several hardware and software modules, as described elsewhere (23, 56, 58–60). In brief, the EUROPattern fluorescent microscope (Figure 2B) is equipped with a 20× objective, two high-resolution cameras, LEDs for fluorescence or transmitted light with a lifespan of >50,000 h, and a matrix code reader for slide identification. The slide magazine has a loading capacity of 500 reaction fields that can be processed within 2.5 h (18 s per analysis). The digital images undergo positive/negative classification by the EUROpattern software, capable of discriminating homogeneous, centromeres, speckled, nuclear dots, nucleolar, nuclear membrane, cytoplasmic, and negative/unspecific patterns (Figure 3). Mixed patterns with varying antibody titers can also be identified. In samples classified as positive, interpretation of the fluorescence pattern is based on the *k*-nearest neighbor algorithm, comparing the image features with a reference database based on more than 5,000 images (115,000 cell references). If a patient sample is analyzed in different dilutions, EUROPattern merges all images into one report containing the proposed pattern/s, titer/s, and the corresponding confidence value/s (Figure 1). Results must be confirmed or may be modified by laboratory personnel, either one by one (positive samples) or batch-wise (negative samples). The EUROPattern graphical user interface is incorporated into the superordinate laboratory management software EUROLabOffice, which allows the EUROPattern system to exchange data with the LIS. Additionally, EUROLabOffice is capable of compiling worklists, interconnecting with other laboratory devices (e.g., Sprinter XL), consolidating the results of different techniques (IFA, ELISA, immunoblot) into one report per patient, and paperless data archiving.

The EUROPattern microscope and software, in combination with the IFA 40: HEP-20-10 EUROPattern assay, has received FDA 510(*k*) clearance (No. k141827).

### Evaluation Criteria

The purpose of this study was to evaluate a new automated ANA IIF protocol (Sprinter/EUROPattern system) as an alternative for a method that was discontinued. Some criteria were considered to evaluate the new IIF protocol: FDA-approved system, automated platform, high-throughput, positive ID throughout the process, workflow compatibility with 8 h shift, LIS interface, reliable pattern recognition, and batch reporting of negatives.

### Statistical Methods

Statistical analyses were performed using GraphPadPrism 6 (GraphPad Software Inc., La Jolla, San Jose, CA, USA). The degree of inter-rater agreement between visual and automated antibody pattern interpretation was assessed by the percentage of concordance and by kappa coefficients. According to Altmann (61), kappa (κ) values were interpreted as follows: ≤0.20 poor, 0.21–0.40 fair, 0.41–0.60 moderate, 0.61–0.80 good, and 0.81–1.00 very good agreement. Confidence intervals (CI 95%) were calculated according to the modified Wald method.

## Results

### Characteristics of HEp-20-10 Slides

Throughout this study, the biochip slides coated with HEp-20-10 cells were of consistent quality. The number of mitotic cells per reaction field exceeded that of standard Hep-2 cells by 10-fold.

### Workflow Evaluation

**Figure 4:**
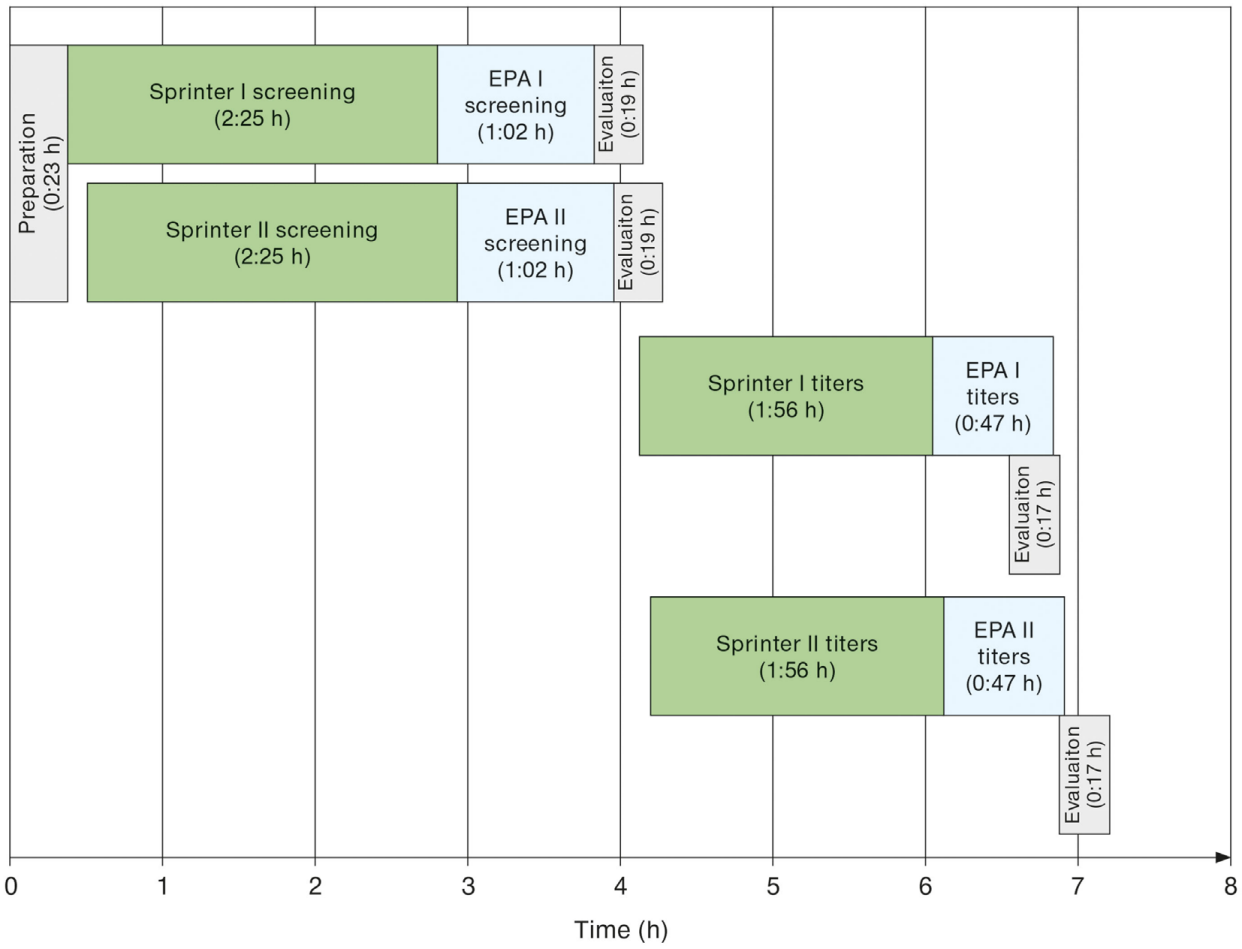
Exemplary schematic workflow of automated anti-nuclear antibodies indirect immunofluorescence as determined at the LabCorp laboratory Dublin (OH, USA) for a daily workload cohort comprising 388 samples. Two Sprinter XL devices were used for sample/slide incubation and two EUROPattern (EPA) devices for image acquisition and interpretation. Initial screening aimed at discriminating negative from positive samples. Only the 194 positive samples were further analyzed for patterns and endpoint titers using three dilutions per sample.

Run times for daily workload cohorts were surveyed at eight LabCorp laboratories using the Sprinter XL for sample preparation and slide processing, followed by EUROPattern-based image acquisition and evaluation. According to the individual number of samples, up to three screening and titer runs were conducted per laboratory, using three dilutions for the determination of endpoint titers in positive samples. The total time requirement was between 06:00 and 07:00 h (Table 1), thus conforming to an 8-h shift. Figure 4 depicts the workflow recorded at the Dublin laboratory, where the processing of 400 samples in two screen and two titer runs took a total time of 07:10 h or approximately 1 min per sample.

**Table 1:** Workflow analysis for anti-nuclear antibodies indirect immunofluorescence testing in average daily workload cohorts at eight LabCorp laboratories (fiscal year 2016).

Laboratory site	Samples (*n*)	Number of devices (*n*)		Runs per day (*n*)		Approximately overall time requirement
				
		Sprinter XL	EUROPattern	Screening	Titer	
Houston, TX, USA	141	1	1	1	1	06:00 h
Tampa, FL, USA	134	1	1	1	1	06:10 h
Dallas, TX, USA	187	1	1	1	1	06:40 h
Phoenix, AZ, USA	231	1	1	1	1	07:10 h
Birmingham, AL, USA	236	2	2	2	1	06:30 h
Burlington, NC, USA	471	2	2	2	2	07:10 h
Dublin, OH, USA	330	2	2	2	2	07:10 h
Raritan, NJ, USA	568	3	3	3	3	07:05 h

### Labor Savings

The demand for labor was compared between the automated Sprinter/EUROPattern system and the manual procedure that was in use. The Sprinter/EUROPattern method comprises two analytical runs: (1) screening for the purpose of positive/negative discrimination at a single dilution, (2) determination of patterns and endpoint titers in positive samples using three dilutions (three-well approach). In contrast, the previous method provided endpoint titers from a single dilution (single-well approach). Calculations for an average of 400 samples per day revealed a total hands-on time of 98 min (01:38 h) for the Sprinter/EUROPattern system, and 355 min (5:55 h) for the previous method, corresponding to a total of 4:20 h labor savings (Table 2).

**Table 2:** Comparison of labor hands-on time between the current LabCorp method (single-well) and the Sprinter/EUROPattern system (two runs, three-well dilution).

	Current method		Sprinter/EUROPattern	
		
	Details	Total time (min)	Details	Total time (min)
**I. Anti-nuclear antibodies (ANA) indirect immunofluorescence screening (on average 400 screens per day, single dilution)**				
Set-up	7 s/sample, manual	47	5.8 s/sample, incl. reagents	39
Pipetting	7.7 s/sample, manual	51	Sprinter XL	0
Washing	10 s/slide, manual	6	Sprinter XL	0
Conjugate	16 s/slide, manual	9	Sprinter XL	0
Washing	10 s/slide, manual	6	Sprinter XL	0
Coverslip	10 s/slide, manual	6.7	6 s/slide (titerplane)	4.0
Slide evaluation	Read in dark room: negative 7.5 s, positive 30 s, mixed positive 45 s	94	Read and release negatives on computer: negative 3 s, borderline 20 s; positives: titer estimation (II)	18
Slide manipulation, focus, writing results	15.2 s/slide, manual	101	EUROPattern	0
Clean-up		5		5
Result entry into computer		30	EUROPattern	0
Total labor for screens (min)		355		65

**II. ANA IIF titers (on average 100 titers per day, three dilutions)**				
Set-up		0	2.5 s/sample, incl. reagents	4
Pipetting		0	Sprinter XL	0
Washing		0	Sprinter XL	0
Conjugate		0	Sprinter XL	0
Washing			Sprinter XL	0
Coverslip		0	6 s/slide (titerplane)	1
Read slides		0	Read slides on computer: positive titer = 10.5 s/patient (all titers displayed on 1 screen)	18
Slide manipulation, focus, writing results		0	EUROPattern	0
Clean-up		0	Daily maintenance, shutdown	10
Result entry into computer		0		0
Total labor for titer (min)		0		33

**III. ANA IIF screening and titers**				
Total labor hands-on time (min)		355		98
Savings EUROPattern (min)				257 (4 h 17 min)

If a single-well approach was used for each of the methods, the labor hands-on time would amount to 82 min (1:22 h) for the Sprinter/EUROPattern system and 355 min (5:55 h) for the current method. The difference in labor savings between the Sprinter/EUROPattern three-well and single-well approach was only 16 min for an 8-h shift (Table 3).

**Table 3:** Comparison of labor hands-on time between the current LabCorp method (single-well) and the Sprinter/EUROPattern system (single-well).

	Current method		Sprinter/EUROPattern	
	Details	Total time (min)	Details	Total time (min)
**I. Anti-nuclear antibodies (ANA) indirect immunofluorescence (IIF) screening (on average 400 screens per day, single dilution)**				
Set-up	7 s/sample, manual	47	5.8 s/sample, incl. reagents	39
Pipetting	7.7 s/sample, manual	51	Sprinter XL	0
Washing	10 s/slide, manual	6	Sprinter XL	0
Conjugate	16 s/slide, manual	9	Sprinter XL	0
Washing	10 s/slide, manual	6	Sprinter XL	0
Coverslip	10 s/slide, manual	6.7	6 s/slide (titerplane)	4.0
Slide evaluation	Read in dark room: negative 7.5 s, positive 30 s, mixed positive 45 s	94	Read and release negatives on computer: negative 3 s, borderline 20 s; positives 10.5 s	34
Slide manipulation, focus, writing results	15.2 s/slide, manual	101	EUROPattern	0
Clean-up		5		5
Result entry into computer		30	EUROPattern	0
Total labor for screens (min)		355		82

**II. ANA IIF single-well analysis**				
Total labor hands-on time (min)		355		82
Savings EUROPattern (min)				273 (4 h 33 min)

### Evaluation of ANA Patterns and Titers

Diagnostic performance for ANA patterns and endpoint titers using the EUROPattern Suite was evaluated using 176 positive and 97 negative samples. The results were compared to the PolyTiter system with or without a technologist reading the slides for both EUROPattern and PolyTiter systems. The number of times the technologist agreed with the initial instrument call for pattern or titer is expressed in % (Table 4).

**Table 4:** Agreement (positive/negative results) between EUROPattern, the current LabCorp method, and technologist reading.

Specimens	*n*	Sample agreement (95% CI)		
		
		EUROPattern vs. current method	Technologist vs. current method	EUROPattern vs. technologist
Homogenous pattern	68	79.4% (68.2–87.4%)	83.8% (73.1–90.9%)	92.6% (83.5–97.2%)
Granular pattern	41	97.6% (86.3–100%)	100% (89.8–100%)	97.6% (86.3–100%)
Centromere pattern	20	95.0% (74.6–100%)	95.0% (74.6–100%)	100% (81.0–100%)
Nucleolar pattern	22	63.6% (42.9–80.4%)	81.8% (60.9–93.3%)	86.4% (65.8–96.1%)
Mixed patterns	25	92.0% (73.9–98.9%)	92.0% (73.9–98.9%)	100% (84.2–100%)

Positive agreement	176	85.2% (79.2–89.8%)	89.8% (84.3–93.5%)	94.9% (90.4–97.4%)
Negative agreement	97	96.9% (90.9–99.3%)	96.9% (90.9–99.3%)	96.9% (90.9–99.3%)
Overall agreement	273	89.4% (85.1–92.5%)	92.3% (88.5–95.0%)	95.6% (92.4–97.6%)
κ-Value		0.780 (0.705–0.854)	0.838 (0.772–0.904)	0.905 (0.853–0.958)

Positive/negative classification by EUROPattern and technologist reading with the PolyTiter system without technologist demonstrated a total agreement rate of 89.4% (κ = 0.780) (Table 4, EUROPattern vs. current method) and 92.3% (κ = 0.838) when technologist was reading slides on EUROPattern Suite vs. the PolyTiter (Table 4, Technologist vs. current method). Highest overall agreement of 95.6% (κ = 0.905) was found when we compared the reading between EUROPattern and a technologist (Table 4, EUROPattern vs. technologist). In each pattern group (except for the granular group), the highest degree of positive agreement was found between EUROPattern and technologist reading, declining in the following order: centromere and mixed patterns (100%), granular patterns (97.6%), homogenous patterns (92.6%), and nucleolar patterns (86.4%) (Table 4).

Pattern assignment using the PolyTiter system showed similar concordance rates for EUROPattern (68.4–100%) and for PolyTiter reading (76.3–100%), with least pattern matches observed among samples with nucleolar or mixed patterns. Endpoint titer agreement (within ±1 dilutions) with the current method varied depending on the pattern type between 5.0 and 72.1% by the EUROPattern Suite and between 35.0 and 73.5% by technologist reading, with lowest rates obtained in the centromere pattern group. High pattern correlation (80.6–97.5%) in combination with the highest endpoint titer agreement rates (71.0–93.8%) were found when comparing EUROPattern vs. technologist reading, indicating that the three-well approach provides higher overall accuracy (Table 5). The PolyTiter method (single-well approach) is at disadvantage when evaluating mixed patterns because it only distinguishes between two patterns.

**Table 5:** Pattern and titer agreement between EUROPattern, the current LabCorp method, and technologist reading.

Specimens	*n*	EUROPattern vs. current method		Technologist vs. current method		EUROPattern vs. technologist	
				
		Pattern	Titer^a,b^	Pattern	Titer^b^	Pattern	Titer^a,b^
Homogenous pattern	68	85.3%	72.1%	85.3%	73.5%	97.5%	93.8%
Granular pattern	41	92.7%	61.0%	92.7%	63.4%	90.7%	85.2%
Centromere pattern	20	100%	5.0%	100%	35.0%	80.6%	74.2%
Nucleolar pattern	22	68.2%	59.1%	77.3%	59.1%	80.6%	71.0%
Mixed patterns	25	68.4%	52.6%	76.3%	55.3%	89.7%	84.6%

*^a^Endpoint titers by EUROPattern were based on a three-well dilution protocol (1:80, 1:320, 1:1,280)*.

*^b^Titer agreement within ±1 dilution*.

If patterns and titers were determined at a single dilution (1:80), 100% correlation was observed between EUROPattern and technologist reading. In contrast, the correlation values between EUROPattern and the PolyTiter method were lower (pattern 75.3%, titer 57.1%) (Table 6).

**Table 6:** Comparison of single-well analysis between EUROPattern and the current LabCorp method or technologist reading.

Dilution	*n*	EUROPattern vs. current method		EUROPattern vs. technologist	
			
		Pattern	Titer^a^	Pattern	Titer^a^
1:40	179	73.3%	59.5%	N/A	N/A
1:80	115	75.3%	57.1%	100%	100%

*^a^Titer agreement within ±1 dilution*.

## Discussion

This study examined the workflow and performance characteristics of the automated Sprinter/EUROPattern IIF system as an alternative to the two methods described herein. It was not the purpose of the study to evaluate the diagnostic performance of the Sprinter/EUROPattern IIF system, therefore, we did not use well-defined characterized patient population, such as (1) autoimmune rheumatic disease patient cohort, (2) non-ARD diseased cohort, and (3) healthy control group in our study, but just negative or positive serum specimen for ANA. Overall, the evaluation criteria for a new automated ANA IIF approach (see Evaluation Criteria) were either met or exceeded. The automated approach completed the daily workload within an 8-h shift and reduced the labor hands-on time for screening and titer runs by more than 4 h. Applying automation in a single-well approach resulted in further labor savings of 16 min. However, this slight reduction should not justify an overall application of the single-well approach considering the associated risk of interferences (masking, prozone effect) and reduced accuracy. Longer walk-away times may contribute to greater laboratory productivity with the gain in higher throughput. In addition, the Sprinter/EUROPattern system is less prone to error in barcode-based sample/slide identification and the use of positive patient IDs throughout the process. The graphical user interface displays all results and the corresponding images, allowing for fast interactive validation of individual positive or batches of negative reports. Since the software-proposed results require verified (or possibly modified) by the operator, subjectivity cannot be completely removed, but the system has the potential to reach 100% concordance with visual microcopy.

The performance of the EUROPattern Suite is in accordance with the ICAP guidelines (34), including the distinction of several nuclear, but also cytoplasmic patterns on native HEp-20 or HEp-2010 cells, the identification of mixed patterns, and the calculation of semi-quantitative endpoint titers on the basis of several dilutions. Sample titration is highly relevant for the discrimination of mixed ANA patterns (60, 62). Using IIF screening at only a single titer, masked patterns can be missed, resulting in incomplete reporting of diagnostically relevant antibodies. According to Carter et al., distinct masked patterns were observed in 1% (29 out of 3,000) of routine ANA samples (63). Similarly, prozone ANA patterns may be indiscernible if the patient sample is not sufficiently diluted, resulting in false-negative results (36, 37). Thus, systems that provide pattern and titer proposals from single-well estimations may be at a disadvantage.

According to our data, the EUROPattern system provided overall improvement with respect to the recognition of ANA patterns and the determination of endpoint titers. Overall highest correlation values resulted from comparing EUROPattern vs. technologist reading, either based on a three-dilution protocol (patterns, 80.6–97.5%; titers, 71.0–93.8%) or on single-well analysis (patterns, 100%; titers, 100%). Lower correlation values with the current method derived (patterns, 68.2–100%; titers, 5.0–72.1%) may be due to inherent flaws such as the utilization of a speckled standard curve only, the occurrence of masking effects, and standardization of the slide manufacturing process.

These findings are consistent with recent literature revealing good performance characteristics of the EUROPattern Suite. For example, Voigt et al. analyzed a total of 351 serum samples to compare the performance of the EUROPattern software-based evaluation with technologist visual interpretation by expert technologists. They also found 99.4% concordant results for positive/negative discrimination with a sensitivity and specificity of the EUROPattern Suite of 100 and 97.5%, respectively. The agreement in main pattern recognition (including mixed patterns) amounted to 94.0% (56). Yoo et al. used the same approach based on 104 samples, reporting a sensitivity and specificity of 94.3 and 94.1%, respectively, and concordance in negative/positive classification of 94.2%. Matching of major patterns occurred in 83.7% of samples with simple and 95.2% with mixed ANA patterns. Comparison of simple pattern titers revealed 82.9% agreement between both methods (59). Tozzoli et al. reported 100% diagnostic sensitivity of the EUROPattern system with reference to manual IFA (16). Bizzaro et al. compared the EUROPattern Suite to five other automated systems (AKLIDES, NOVA View, Zenit G-Sight, Helios, and Image Navigator) using 126 manually pre-characterized sera. This study, which was the first to compare the diagnostic accuracy of six systems for automated ANA-IIF reading on the same series of sera, showed that all systems are able to perform very well the task for which they were created. Overall sensitivity of the six automated systems was 96.7% and overall specificity was 89.2%. Most false negatives were recorded for cytoplasmic patterns, whereas among nuclear patterns those with a low level of fluorescence (i.e., multiple nuclear dots, midbody, and nuclear rim) were sometimes missed. The intensity values of the light signal of various instruments showed a good correlation with the titer obtained by manual reading (Spearman’s rho between 0.672 and 0.839; *P* < 0.0001 for all the systems). Imprecision ranged from 1.99 to 25.2% and, for all the systems, it was lower than that obtained by the manual IIF test (39.1%). The accuracy of pattern recognition, which is for now restricted to the most typical patterns (homogeneous, speckled, nucleolar, centromere, multiple nuclear dots, and cytoplasmic) was limited, ranging from 52 to 79%. The systems demonstrated overall concordance rates for the classification of positive and negative results of 93.7–96.8% (EUROPattern: 93.7%), and correct pattern assignment in 52–79% (EUROPattern: 79%) (30).

Like similar automated instruments for ANA reading and interpretation, the EUROPattern Suite is a closed system, i.e., neither microscope nor software is interchangeable with other analogous devices (23, 30). The EUROPattern Suite is restricted to the use of Euroimmun IIF kits as this is the only way to guarantee high quality of results. For EUROPattern-based evaluation, Euroimmun offers not only slides coated with HEp-2 or HEp-20-10 cells, but also several other cell substrates for other diagnostic purposes (e.g., *Crithidia luciliae*, ethanol-fixed and formalin-fixed human granulocytes, transfected cells, and infected cells). Noteworthy, IFA40: HEp-20-10 kits contribute to the standardization of EUROPattern-based ANA testing. Constant quality of the substrate is ensured by quality control measures throughout the production of the HEp-20-10-coated biochips. Reliability of the assay has been demonstrated by validation studies (57).

In conclusion, the EUROPattern Suite, along with the Sprinter IIF slide processor, is a fully automated solution for ANA IIF testing on HEp-20-10 cells, allowing laboratories to perform testing on hundreds of samples per day. The Sprinter/EUROPattern system enables substantially reduced hands-on time and high correlation with technologist visual IIF microscopy, thus supporting high throughput, labor savings, and standardized operations.

## Ethics Statement

In accordance with the Declaration of Helsinki (1964) ethical guidelines, samples were blinded for analysis to maintain confidentiality. The study protocol was determined to be exempt by the Institutional Review Board.

## Author Contributions

All authors designed, analyzed data, and wrote the manuscript.

## Conflict of Interest Statement

Reagent and instrumentation were provided by Euroimmun for validation purpose. The reviewer OC and handling Editor declared their shared affiliation.
